# Sex-related differences in the association between frailty and dietary consumption in Japanese older people: a cross-sectional study

**DOI:** 10.1186/s12877-019-1229-5

**Published:** 2019-08-05

**Authors:** Koji Shibasaki, Shin Kei Kin, Shizuru Yamada, Masahiro Akishita, Sumito Ogawa

**Affiliations:** 1 Department of Geriatric Medicine, Graduate School of Medicine, The University of Tokyo, 7-3-1 Hongo, Bunkyo-ku, Tokyo, Japan; 2 Department of Physical Therapy, Health Science University, 7178 Kodachi, Fujikawaguchiko-town, Yamanashi, Japan; 3 Komagane-kogen Ladies Clinic, 759-195, Akaho, Komagane-city, Nagano Japan

**Keywords:** Dietary consumption, Frailty, Nutrition, Older adults, Sex-related differences

## Abstract

**Background:**

Female sex is an important factor predisposing individuals to frailty. Appropriate nutrition is one of the most effective ways to prevent older adults from developing frailty; Sex-related differences have also been detected in the association between nutritional intervention and health-related outcomes. However, few studies have discussed these sex-related differences. The aim of the present study was to investigate the sex-related differences in the association between frailty and dietary consumption.

**Methods:**

We conducted a cross-sectional study which investigated community-dwelling older adults aged ≥65 years. We surveyed age, sex, body mass index, family arrangement (living alone, living with a partner or living with parent(s) and/or child (ren)), dietary consumption and frailty status. Dietary consumption was surveyed using a food frequency questionnaire that included 13 major food categories (fish, meat, eggs, dairy products, soybean products, vegetables, seaweeds, potatoes, fruits, fats or oils, snacks, salty foods and alcohol). Frailty was defined by the Kihon Checklist score. The Kihon Checklist is composed of 25 simple yes/no questions, and it has been validated as a metric for frailty. A higher score indicates a greater degree of frailty. Multinomial regression analysis was performed to clarify the association between frailty and dietary consumption for each sex.

**Results:**

We analyzed 905 older adults (420 (46.4%) were male). After adjusting for cofounders, a low frequency of meat consumption (less than twice/week) was associated with a high prevalence of frailty in men (odds ratio: 2.76 (95%CI: 1.12–6.77), *p* = 0.027). In contrast, in women, low frequencies of consumption of fish, meat, vegetables, potatoes and snacks were associated with a higher prevalence of frailty compared with those who consumed foods from those categories daily (odds ratios: fish 2.45 (1.02–5.89), *p* = 0.045; meat 4.05 (1.67–9.86), *p* = 0.002; vegetables 5.03 (2.13–11.92), *p* < 0.001; potatoes 3.84 (1.63–9.05), p = 0.002; snacks 2.16 (1.02–4.56), *p* = 0.043).

**Conclusions:**

More food categories were associated with frailty in women than in men. Nutritional intervention to prevent frailty is presumably more effective for women than for men.

**Electronic supplementary material:**

The online version of this article (10.1186/s12877-019-1229-5) contains supplementary material, which is available to authorized users.

## Background

Japan is facing a super-aged society, and the number of older people with frailty is increasing. Frailty leads not only to disability, long-term care and higher mortality but also to increased health-related costs. Preventing frailty in older people is an urgent task in Japan and other developed countries. Currently, several frailty criteria exist, such as those proposed by Fried L et al. or Rockwood K et al. [1, 2]. These criteria have shown an association between frailty and high mortality rates. Fried L et al. showed greater frailty in those who were older age, female, and African American, with less education, lower income, poorer health, and higher rates of comorbid chronic disease. Rockwood K et al. also identified that people who were older age, female, cognitively impaired and incontinent were more frail. These two reports both showed an association between frailty and female sex. Furthermore, a systematic review and meta-analysis showed consistent results [3].

One of the most effective ways to prevent frailty is to consume appropriate nutrition. In addition to the intake of protein, the consumption of meat, fish, soybeans, milk, fruit and vegetables decreases the prevalence of frailty. The Mediterranean diet is also known to prevent frailty in older people [4–6]. Houston DK et al. reported in their three-year cohort study that older men and women in the highest quintile in terms of protein intake lost approximately 40% less lean mass and appendicular lean mass than those in the lowest quintile in terms of protein intake [7]. The Framingham Third Generation Study also showed a significant association between high levels of protein intake and higher appendicular lean mass and quadriceps strength [8]. Moreover, the intake of protein and vitamin D demonstrated protective effects against frailty. There is a growing body of evidence that links frailty to nutrition and dietary intake in older adults. However, sex-related differences in the association between frailty and dietary consumption are under discussion. A multicenter cross-sectional study that recruited 2108 older women aged ≥65 years reported that high protein intake was associated with a low prevalence of frailty [9]. However, Shikany et al. showed no association between protein intake and frailty in 5925 older men aged ≥65 years [10].

Previous studies have shown sex-related differences in the association between dietary consumption and health-related outcomes such as cardiovascular disease and mortality [11–15]. Fiber intake was reported to decrease mortality only in females and not in males [14]. The consumption of milk and dairy products was reported to be associated with high mortality rates only in females and not in males [12]. In addition, higher levels of consumption of red meat and processed meat were associated with higher mortality rates in females than in males [15].

However, few studies have focused on sex-related differences in dietary consumption in frail older adults. The aim of the present study was to investigate sex-related differences in the association between frailty and dietary consumption.

## Methods

### Participants and settings

This study protocol was approved by the institutional review board of the University of Tokyo. Written informed consent was obtained from all participants or their families.

We analyzed 905 community-dwelling older adults in this cross-sectional study. All participants lived in the village of Minami-Minowa, Nagano Prefecture, Japan. We sent questionnaires through the mail to all participants in 2015 with simple instructions who met the inclusion criteria. The inclusion criteria were those aged 65 years or older and participants who did not need to use long-term care insurance. A proxy was allowed to answer the questionnaire, but we excluded functionally dependent older adults (i.e., those requiring nursing care provided by long-term care insurance). We did not conduct a pilot survey because we used a previously validated questionnaire. One thousand two hundred twenty-six participants responded, and 905 participants out of the 1226 who provided written informed consent were analyzed in this study.

### Kihon checklist, frailty definition

The Kihon Checklist is one of the most popular questionnaires for older adults in Japan (Table 1). Kihon means basic or basically in Japanese. The Japanese Ministry of Health, Labor and Welfare conducted a survey with the Kihon Checklist to identify all older adults living in Japan at risk of requiring care or support to prevent older adults from developing disability or needing long-term care. The Kihon Checklist was translated into English and validated as a metric for frailty [16, 17]. It comprises 25 simple yes/no questions, with each question allocated 1 point. A “no” answer in question numbers 1, 2, 3, 4, 5, 6, 7, 8, 16, and 19 is equal to 1 point each. A “yes” answer in these questions is equal to 0 point each. Meanwhile, a “yes” answer in question numbers 9, 10, 11, 12, 13, 14, 15, 17, 18, 20, 21, 22, 23, 24, and 25 equals to 1 point each. A “no” answer in these questions in equal to 0 point each. The Kihon checklist score ranges from 0 to 25, with a higher score indicating a greater degree of frailty. Satake et al. reported that scores from 0 to 3, 4 to 7 and 8 or greater indicated normal (nonfrailty), prefrailty and frailty, respectively [16]. We defined participants as belonging to normal, prefrail and frail categories based on this report.

**Table 1:** Kihon Checklist and differences according to sex

	Men, n (%)		Women, n (%)	
	YES	NO	YES	NO
1. Do you go out by bus or train by yourself?	336 (80.0)	**84 (20.0)**	389 (80.2)	**96 (19.8)**
2. Do you go shopping to buy daily necessities by yourself?	401 (95.5)	**19 (4.5)**	461 (95.1)	**24 (4.9)**
3. Do you manage your own deposits and savings at the bank? *	375 (89.3)	**45 (10.7)**	453 (93.4)	**32 (6.6)**
4. Do you sometimes visit your friends?	353 (84.0)	**67 (16.0)**	420 (86.6)	**65 (13.4)**
5. Do you turn to your family or friends for advice?	390 (92.9)	**30 (7.1)**	457 (94.2)	**28 (5.8)**
6. Do you normally climb stairs without using a handrail or wall for support? *	331 (78.8)	**89 (21.2)**	315 (64.9)	**170 (35.1)**
7. Do you normally stand up from a chair without any aids? *	385 (91.7)	**35 (8.3)**	406 (83.7)	**79 (16.3)**
8. Do you normally walk continuously for 15 min?	368 (87.6)	**52 (12.4)**	407 (83.9)	**78 (16.1)**
9. Have you experienced a fall in the past year?	**74 (17.6)**	346 (82.4)	**93 (19.2)**	392 (80.8)
10. Do you have a fear of falling while walking? *	**99 (23.6)**	321 (76.4)	**211 (43.5)**	274 (56.5)
11. Have you lost 2 kg or more in the past 6 months?	**38 (9.0)**	382 (91.0)	**55 (11.3)**	430 (88.7)
12. Height: cm, weight: kg, BMI: kg/m^2^ If BMI is less than 18.5, this item is scored.	**23 (5.5)**	397 (94.5)	**35 (7.2)**	450 (92.8)
13. Do you have any difficulties eating tough foods compared to 6 months ago?	**71 (16.9)**	349 (83.1)	**100 (20.6)**	385 (79.4)
14. Have you choked on your tea or soup recently?	**74 (17.6)**	346 (82.4)	**88 (18.1)**	397 (81.9)
15. Do you often experience having a dry mouth?	**96 (22.9)**	324 (77.1)	**100 (20.6)**	385 (79.4)
16. Do you go out at least once a week? *	404 (96.2)	**16 (3.8)**	441 (90.9)	**44 (9.1)**
17. Do you go out less frequently compared to last year? *	**58 (13.8)**	362 (86.2)	**95 (19.6)**	390 (80.4)
18. Do your family or your friends point out your memory loss? e.g., “You ask the same question over and over again.”	**57 (13.6)**	363 (86.4)	**57 (11.8)**	428 (88.2)
19. Do you make a call by looking up phone numbers?	400 (95.2)	**20 (4.8)**	467 (96.3)	**18 (3.7)**
20. Do you find yourself not knowing today’s date?	**73 (17.4)**	347 (82.6)	**92 (19.0)**	393 (81.0)
21. In the last 2 weeks, have you felt a lack of fulfillment in your daily life?	**44 (10.5)**	376 (89.5)	**48 (9.9)**	437 (90.1)
22. In the last 2 weeks, have you felt a lack of joy when doing the things you used to enjoy?	**28 (6.7)**	392 (93.3)	**44 (9.1)**	441 (90.9)
23. In the last 2 weeks, have you felt difficulty in doing what you could do easily before?	**111 (26.4)**	309 (73.6)	**151 (31.1)**	334 (68.9)
24. In the last 2 weeks, have you felt helpless? *	**68 (16.2)**	352 (83.8)	**55 (11.3)**	430 (88.7)
25. In the last 2 weeks, have you felt tired without a reason?	**88 (21.0)**	332 (79.0)	**103 (21.2)**	382 (78.8)

Values are presented as n (%); bold letter indicates scoring

*p < 0.05, chi-squared test for sex-related differences

### Food frequency questionnaire

The dietary data included the following 13 major food groups: fish, meat, eggs, dairy products, soybean products, vegetables, seaweeds, potatoes, fruits, fats or oils, snacks, salty foods and alcohol. Participants chose from the following 4 frequency categories: daily, 3 to 6 times per week, 1 to 2 times per week or less than once per week. This food frequency questionnaire was validated for use in investigating frailty [18], and similar food frequency questionnaires have been widely applied to investigate the relationship between dietary consumption and mortality, cardiovascular disease and other health-related outcomes [19]. Therefore, we applied this method.

Ten food categories out of 13 were used to calculate dietary variety score [18]. Those ten categories were fish, meat, eggs, dairy products, soybean products, vegetables, seaweeds, potatoes, fruits and fats or oils. The answer ‘Daily’ was scored as 1 point, and 6 times or less per week was scored as 0 for each food category. The maximum score was 10, and a high score indicated high dietary variety.

### The other variables

We also asked participants about their height, weight and family arrangement (participants who lived alone, lived with a partner or lived with parent(s) and/or child (ren)).

### Statistical analyses

Data were analyzed using SPSS software (Ver. 24.0, SPSS Japan Inc., Tokyo, Japan). The participants were divided into the following 3 categories based on their scores on the Kihon Checklist: normal, prefrailty and frailty.

The chi-squared test was used for categorical variables to investigate the relationships between each item of Kihon Checklist and sex-related differences, the prevalence of frailty and sex-related differences, family arrangement and food categories the frequency of consumption of the different categories of food.

The data from 762 participants was used for multinomial regression analyses. Family arrangement data was unknown (missing values) for 143 participants. In the multinomial regression analyses, the data were adjusted for age, body mass index and family arrangement. Odds ratios (OR) indicate the tendency of participants who consume foods at a frequency of 3–6 times/week and less than twice/week to have prefrailty and frailty compared with those who consume these foods daily. For the multinomial regression analyses, the original 4 frequency categories on the food questionnaire were redistributed into 3 categories as follows: daily, 3–6 times per week and fewer than two times per week; this redistribution was performed because the number of participants who answered ‘Less than once per week’ in each food category was extremely low except for dairy products, salty foods and alcohol (fish: 5 (0.6%), meat: 21 (2.3%), eggs: 37 (4.1%), dairy products: 190 (21.0%), soybean products: 15 (1.7%), vegetables: 7 (0.8%), seaweeds: 24 (2.7%), potatoes: 26 (2.9%), fruits: 29 (3.2%), fats or oils: 56 (6.2%), snacks: 85 (9.4%), salty foods: 505 (55.8%) and alcohol: 531 (58.7)). Therefore, we pooled the group that answered 1 to 2 times per week and the group that answered less than once per week.

Two-way analysis of variance (ANOVA) was performed to investigate the interaction between sex and each of the 13 categories of dietary consumption. The Bonferroni method was used for post-hoc tests after two-way ANOVA.

The association between the dietary variety score and frailty was calculated by Kruskal-Wallis analyses and multinomial regression analyses (adjusted for age, body mass index and family arrangement). A *P* value of <0.05 was considered significant.

## Results

**Fig. 1:**
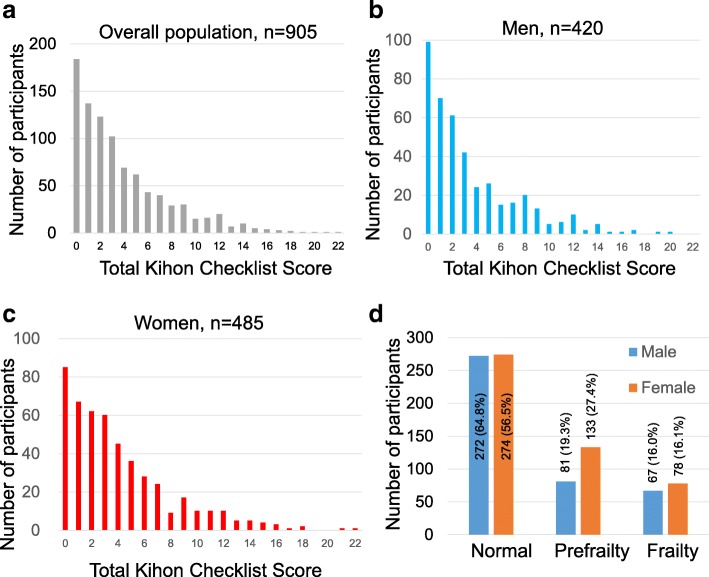
The total Kihon Checklist scores and the prevalence of prefrailty and frailty. **a** The distribution of Kihon Checklist scores. Higher scores indicates greater frailty (range: 0 to 25). **b** The distribution of Kihon Checklist scores in men. **c** The distribution of Kihon Checklist scores in women. **d** The prevalence of prefrailty and frailty based on the Kihon Checklist scores

Of the 1226 participants who responded to the survey, 905 provided written informed consent and were included in the analysis. The participant characteristics are shown in Table 2. Table 1 shows the distribution of Kihon Checklist scores. The average score on the Kihon Checklist was 3.78 ± 3.9 (Fig. 1a, b, c). Significant sex-related differences was detected for 7 items (Question 3, 6, 7, 10, 16, 17 and 24). Women had higher scores than men on five out of those 7 items (Questions 6, 7, 10, 16 and 17). The numbers of normal (nonfrail), prefrail and frail individuals were 546 (60.3%), 214 (23.6%) and 145 (16.0%), respectively. Among men, 272, 81, and 67, were categorized into the normal (nonfrail), the prefrail, and the frail groups, respectively, while 274, 133, and 78 women were categorized to these groups, respectively (Fig. 1d). The prevalence of prefrailty and the prevalence of frailty were significantly higher in women than in men.

**Table 2:** The characteristics of the participants

Variables		Values	Men	Women
The number of participants	n (%)	905	420 (46.4%)	485 (53.6%)
Age	Mean ± SD	75.7 ± 5.0	76.0 ± 5.1	75.5 ± 4.9
Body mass index		22.5 ± 2.9	22.7 ± 2.6	22.2 ± 2.9
Family arrangement				
living alone, n (%)		96 (10.6%)	33 (7.9%)	63 (13.0%)
living with a partner, n (%)		329 (36.4%)	168 (40.0%)	161 (33.2%)
living with parent(s) and/or child (ren), n (%)		337 (37.2%)	138 (32.9%)	199 (41.0%)
unknown, n (%)		143 (15.8%)	81 (19.3%)	62 (12.8%)

**Fig. 2:**
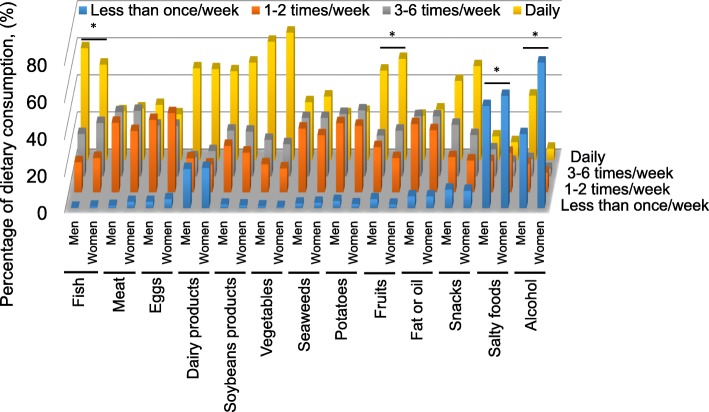
The distribution of answers to the food frequency questionnaire and differences between the sexes. Blue bar, participants who consumed each dietary category less than once/week; Orange bar, 1 to 2 times/week; Gray bar, 3 to 6 times/week; Yellow bar, daily. *p < 0.05, chi-squared test for differences between the sexes

The chi-squared test revealed that men who lived alone ate significantly fewer vegetables (*p* = 0.001), potatoes (*p* < 0.001), and snacks (*p* = 0.005) than those who lived with a partner or with parent(s) or/and child (ren). Women who live alone ate significantly less, meat (*p* = 0.033) and fruit (*p* = 0.025). Compared with women, men consumed significantly more fish, salty food, and alcohol and less fruit (Fig. 2).

As shown in Table 3, after adjusting for age, body mass index and family arrangement in the multinomial regression analyses, older men who consumed meat less than twice per week had a higher prevalence of frailty than those who consumed meat daily (odds ratio: 2.76 (95%CI: 1.12–6.77), *p* = 0.027). We detected a greater number of significant associations between the consumption of dietary elements and frailty in women than in men. Women who consumed fish, meat, vegetables, seaweeds and potatoes less than twice per week had a higher prevalence of prefrailty than those who consumed those dietary elements daily. The odds ratios were as follows: fish 2.12 (1.11–4.05), *p* = 0.023; meat 2.22 (1.18–4.18), *p* = 0.013; vegetables 2.14 (1.05–4.37), *p* = 0.036; seaweeds 2.17 (1.19–3.95), *p* = 0.011; and potatoes 1.96 (1.03–3.74), *p* = 0.040. Women who consumed fish, meat, vegetables, potatoes and snacks less than twice per week had a higher prevalence of frailty (odds ratios: fish 2.45 (1.02–5.89), *p* = 0.045; meat 4.05 (1.67–9.86), *p* = 0.002; vegetables 5.03 (2.13–11.92), *p* < 0.001; potatoes 3.84 (1.63–9.05), p = 0.002; and snacks 2.16 (1.02–4.56), *p* = 0.043). Women who consumed vegetables 3–6 times per week also had a higher prevalence of frailty compared with those who consumed vegetables daily (odds ratio: 3.88 (1.69–8.87), *p* = 0.001).

**Table 3:** Odds ratios for prefrailty and frailty in both men and women

	Prefrailty				Frailty			
	3–6 times/week		Less than twice/week		3–6 times/week		Less than twice/week	
	OR (95%CI)	*p*-value	OR (95%CI)	*p*-value	OR (95%CI)	*p*-value	OR (95%CI)	*p*-value
Men								
Fish	1.13 (0.58–2.19)	0.727	0.91 (0.38–2.19)	0.839	0.66 (0.28–1.58)	0.352	1.94 (0.83–4.56)	0.127
Meat	2.01 (0.90–4.45)	0.087	2.06 (0.93–4.57)	0.075	1.89 (0.76–4.69)	0.172	2.76 (1.12–6.77)	0.027*
Eggs	1.63 (0.75–3.55)	0.221	1.61 (0.79–3.28)	0.189	1.15 (0.50–2.62)	0.744	0.89 (0.42–1.90)	0.772
Dairy products	0.12 (0.02–0.91)	0.040*	0.99 (0.55–1.79)	0.980	1.56 (0.56–4.33)	0.397	1.56 (0.79–3.11)	0.204
Soybeans products	0.87 (0.42–1.81)	0.717	0.89 (0.45–1.78)	0.748	1.20 (0.55–2.60)	0.650	1.03 (0.46–2.30)	0.937
Vegetables	1.28 (0.62–2.62)	0.503	1.32 (0.58–3.00)	0.501	0.97 (0.41–2.28)	0.946	1.85 (0.77–4.42)	0.168
Seaweeds	1.37 (0.64–2.96)	0.420	1.79 (0.88–3.67)	0.110	1.71 (0.72–4.09)	0.227	2.13 (0.94–4.82)	0.070
Potatoes	1.78 (0.80–3.94)	0.155	1.68 (0.77–3.67)	0.191	1.67 (0.71–3.96)	0.243	1.59 (0.68–3.72)	0.284
Fruits	0.82 (0.38–1.79)	0.624	1.33 (0.69–2.59)	0.395	1.16 (0.50–2.68)	0.722	1.75 (0.81–3.76)	0.154
Fat or oil	1.67 (0.69–4.03)	0.251	2.59 (1.14–5.88)	0.022*	0.87 (0.36–2.14)	0.766	1.56 (0.69–3.53)	0.281
Snacks	0.90 (0.46–1.78)	0.770	0.98 (0.48–2.00)	0.951	0.50 (0.22–1.14)	0.099	1.19 (0.56–2.56)	0.648
Salty foods	2.24 (0.53–9.45)	0.273	2.81 (0.81–9.74)	0.104	0.64 (0.20–2.06)	0.460	0.49 (0.19–1.26)	0.138
Alcohol	1.70 (0.65–4.47)	0.282	1.54 (0.81–2.93)	0.185	0.87 (0.22–3.41)	0.843	1.58 (0.77–3.23)	0.211
Women								
Fish	1.08 (0.62–1.90)	0.785	2.12 (1.11–4.05)	0.023*	1.51 (0.73–3.09)	0.264	2.45 (1.02–5.89)	0.045*
Meat	1.30 (0.70–2.42)	0.411	2.22 (1.18–4.18)	0.013*	2.21 (0.93–5.26)	0.073	4.05 (1.67–9.86)	0.002*
Eggs	1.73 (0.87–3.46)	0.118	1.70 (0.9–3.22)	0.101	0.59 (0.25–1.39)	0.225	0.89 (0.42–1.86)	0.747
Dairy products	0.69 (0.32–1.48)	0.341	1.06 (0.62–1.79)	0.839	1.02 (0.38–2.75)	0.968	1.76 (0.88–3.51)	0.111
Soybean products	1.39 (0.77–2.53)	0.274	1.63 (0.89–2.98)	0.114	2.03 (0.96–4.27)	0.062	1.59 (0.70–3.64)	0.270
Vegetables	1.86 (0.96–3.59)	0.064	2.14 (1.05–4.37)	0.036*	3.88 (1.69–8.87)	0.001*	5.03 (2.13–11.92)	< 0.001*
Seaweeds	1.39 (0.76–2.53)	0.290	2.17 (1.19–3.95)	0.011*	0.73 (0.33–1.62)	0.443	1.46 (0.68–3.11)	0.328
Potatoes	1.35 (0.72–2.54)	0.353	1.96 (1.03–3.74)	0.040*	1.45 (0.60–3.49)	0.405	3.84 (1.63–9.05)	0.002*
Fruits	0.97 (0.54–1.76)	0.926	1.44 (0.78–2.66)	0.243	1.54 (0.73–3.23)	0.258	1.31 (0.53–3.21)	0.560
Fat or oil	1.17 (0.62–2.23)	0.629	1.82 (1.00–3.3)	0.050	1.30 (0.60–2.83)	0.503	1.00 (0.45–2.22)	0.991
Snacks	0.77 (0.41–1.43)	0.401	0.90 (0.51–1.61)	0.726	1.38 (0.61–3.15)	0.443	2.16 (1.02–4.56)	0.043*
Salty foods	2.13 (0.63–7.22)	0.224	1.31 (0.55–3.12)	0.538	1.02 (0.19–5.34)	0.982	0.91 (0.32–2.54)	0.850
Alcohol	0.45 (0.07–2.94)	0.408	0.93 (0.32–2.65)	0.887	1.43 (0.16–12.41)	0.747	1.21 (0.29–5.13)	0.794

OR: Odds ratios, Multinomial regression analyses were adjusted for age, body mass index and family arrangement. *p < 0.05

OR indicate the tendency of participants who consume foods at a frequency of 3–6 times/week and less than twice/week to have prefrailty and frailty compared with those who consume these foods daily

Two-way ANOVA revealed significant interaction between sex and vegetable consumption in participants who lived with a partner. Post-hoc test indicated that frailty was significantly less prevalent in women who consumed vegetables daily compared with those who consumed vegetables less than twice /week (*p* < 0.001). Meanwhile, although there tended to be an interaction between sex in fish and dairy products in participants who lived alone and between sex and soy bean products in participants who lived with a partner, such interaction was not statistically significant (Additional file 2: Table S1).

The dietary variety score was lower in frail men and women than in normal participants, but the differences were not significant (Men: normal; 4.3 ± 2.7, prefrailty; 3.7 ± 2.6, frailty; 3.6 ± 2.5, *p* = 0.057, Women: normal; 4.4 ± 2.7, prefrailty; 4.0 ± 2.6, frailty; 3.9 ± 2.4, *p* = 0.134). The multinomial regression analyses showed that frail and prefrail women had significantly lower dietary variety scores than nonfrail women, but the same trend was not observed in men (odds ratio, women: prefrailty; 0.879 (95%CI; 0.793–0.975), *p* = 0.015, frailty; 0.835 (95%CI; 0.727–0.959), *p* = 0.011. Men: prefrailty; 0.911 (95%CI; 0.806–1.029), p = 0.134, frailty; 0.872 (95%CI; 0.759–1.002), *p* = 0.053) (Table 4).

**Table 4:** Dietary variety score

	Normal	Prefrail	Frail
Men			
Dietary variety score, mean ± SD	4.3 ± 2.7	3.7 ± 2.6	3.6 ± 2.5
Odds ratios, (95%CI), p-value	–	0.911 (0.806–1.029), *p* = 0.134	0.872 (0.759–1.002), *p* = 0.053
Women			
Dietary variety score, mean ± SD	4.4 ± 2.7	4.0 ± 2.6	3.9 ± 2.4
Odds ratios, (95%CI), *p*-value	–	0.879 (0.793–0.975), *p* = 0.015	0.835 (0.727–0.959), *p* = 0.011

## Discussion

In summary, the present study demonstrated that the prevalence of prefrailty and frailty was higher in women than in men. Sex-related differences in the association between frailty and dietary consumption were clarified. A higher prevalence of frailty was detected in men who consumed less meat than in men who consumed more meat, and a higher prevalence of frailty was detected in women who consumed less fish, meat, vegetables, potatoes and snacks than in women who consumed those dietary elements with greater frequency. A low dietary variety score was significantly associated with frailty only in women.

Previous reports revealed an association between frailty and dietary consumption without considering sex-related differences. In their systematic review of longitudinal studies, Feng Z et al. reported that lower levels of consumption of fruits and vegetables were significantly associated with frailty [4]. Moreover, low protein intake has been reported to be connected not only to frailty but also to low lean mass, appendicular lean mass and muscle strength [7, 8, 20]. A Mediterranean diet is also known to prevent frailty. However, only a few studies have considered sex-related differences, despite the fact that sex-related differences in the association between the prevalence of frailty and dietary consumption have been consistently demonstrated. A study that analyzed only men showed no association between protein intake and frailty [10]. However, another study demonstrated a significant association between protein intake and frailty [9].

There are several reasons why sex-related differences in the association between dietary consumption and frailty are detected. First, Sharkey JR and colleagues discussed that compared with men, women require more calcium and vitamin D, which particularly affect bone development, bone maintenance and muscular function [21]. Additionally, falls, osteoporosis and fractures are more prevalent in older women than in older men. For these reasons, the association between frailty and the frequency of consumption of fish (which is rich in calcium and vitamin D) was detected in women but not in men. Our data showed that more women than men had a fear of falling while walking. Second, chronic inflammation may affect sex-related differences in the association between frailty and dietary consumption. Canon ME and colleagues reported that decreases in sex hormones in postmenopausal women lead to chronic inflammation and elevated levels of C-reactive protein and interleukin-6 [22]. Chronic inflammation promotes proteolysis and muscle catabolism; therefore, chronic inflammation may help explain the association, especially in women, between the inadequate intake of protein such as meat and fish and frailty. Third, we should take into consideration sex-related differences in the relationship between comorbidities and dietary consumption because comorbidities constitute one of the most strongly predictive factors for frailty [1]. Previous reports demonstrated the relationships between dietary consumption and comorbidities such as cardiovascular disease, cerebrovascular disease and cancer as well as the relationship between dietary consumption and mortality. Du et al. followed 451,665 participants, and they showed that the daily consumption of fresh fruits reduced systolic blood pressure, blood glucose, ischemic stroke, hemorrhagic stroke, major cardiovascular disease and death from cardiovascular causes [19]. In other studies, a higher incidence of cardiovascular disease was detected in participants with lower intake levels of vegetables [23, 24], fiber [14], protein [25], and white meat [15] and those with higher intake levels of milk [12, 26], red meat, and processed meat [15, 27]. Although some studies could not demonstrate or did not mention sex-related differences in the association between dietary consumption and comorbidities [22–24, 27, 28], many studies indicated significantly a higher incidence of comorbidities in women than in men [12, 14, 15, 25, 26]. We hypothesize that greater frequency of consumption of meat, fish and vegetables may prevent older women from developing comorbidities, resulting in protection against frailty.

The total score of Kihon Checklist was significantly higher in women than in men particularly in physical questionnaires such as climbing stairs, stand up from a chair and fear of falling. Also, the odds ratios in the association between dietary consumption and frailty were higher in women than men. These results are probably because low meat and fish consumption have an effect on higher prevalence of physical frailty in women compared with men. Our results are consistent with previous studies that indicate higher protein intake is associated to lower risk of frailty only in women [29].

In this study, there were three food categories that indicated inconsistent results, namely, dairy products, fat or oil in men, and seaweeds in women. These three food categories were significantly associated with prefrailty, but not frailty, and this might be attributed to two reasons. First, these results arose from the distribution of the participants that consumed each food category. For example, only 2 prefrailty participants consumed dairy products 3–6 times/week (Additional file 1: Figure S1 g), and thus statistical error might have occurred. Second, there were more participants with prefrailty than those with frailty, and statistical significance tended to be more detected in the prefrailty than in the frailty group.

Some limitations of this study should be mentioned. First, the present study is a cross-sectional study and cannot provide direct evidence of causality. It is possible to interpret that frail older men and women cannot consume more fish, meat and vegetables. Second, the mortality and the incidence of comorbidities are different between those who consume red meat and those who consume white meat and between those who consume animal protein and those who consume vegetable protein. The type of meat or protein being consumed by older men and women should be considered. Third, because the Kihon Checklist is based on a self-report questionnaire, the results might have been affected by the participant’s memory, and the checklist should thus be adjusted according to the participant’s cognitive function. Finally, we assessed food frequency qualitatively rather than quantitatively. Energy intake should be evaluated to support our results.

## Conclusion

As a conclusion, the present study demonstrated sex-related differences in the association between dietary consumption and frailty. Women were identified as having a greater association between food categories and frailty than men. The consumption of fish, meat, vegetables and potatoes is recommended to prevent frailty in women.

## Additional files

Additional file 1: **Figure S1.** The consumption of each food categories in both men and women. a: Fish (Men), b: Fish (Women), c: Meat (Men), d: Meat (Women), e: Eggs (Men), f: Eggs (Women), g: Dairy products (Men), h: Dairy products (Women), i: Soybeans products (Men), j: Soybeans products (Women), k: Vegetables (Men), l: Vegetables (Women), m: Seaweeds (Men), n: Seaweeds (Women), o: Potatoes (Men), p: Potatoes (Women), q: Fruits (Men), r: Fruits (Women), s: Fat or oils (Men), t: Fat or oils (Women), u: Snacks (Men), v: Snacks (Women), w: Salty foods (Men), x: Salty foods (Women), y: Alcohol (Men), z: Alcohol (Women). The number in each colored bar indicates the number of participants. (PDF 194 kb)

Additional file 2: **Table S1.** Interaction between sex and dietary consumption according to living arrangement (DOCX 23 kb)

## Data Availability

The datasets used and/or analysed during the current study are available from the corresponding author on reasonable request.

## Abbreviations

ANOVA: analysis of variance
OR: odds ratio
