# Supplementary figures and images for: Sex-related differences in the association between frailty and dietary consumption in Japanese older people: a cross-sectional study
Source: BMC Geriatr. 2019 Aug 5;19:211. doi: 10.1186/s12877-019-1229-5 (PMC6683375; doi:10.1186/s12877-019-1229-5)

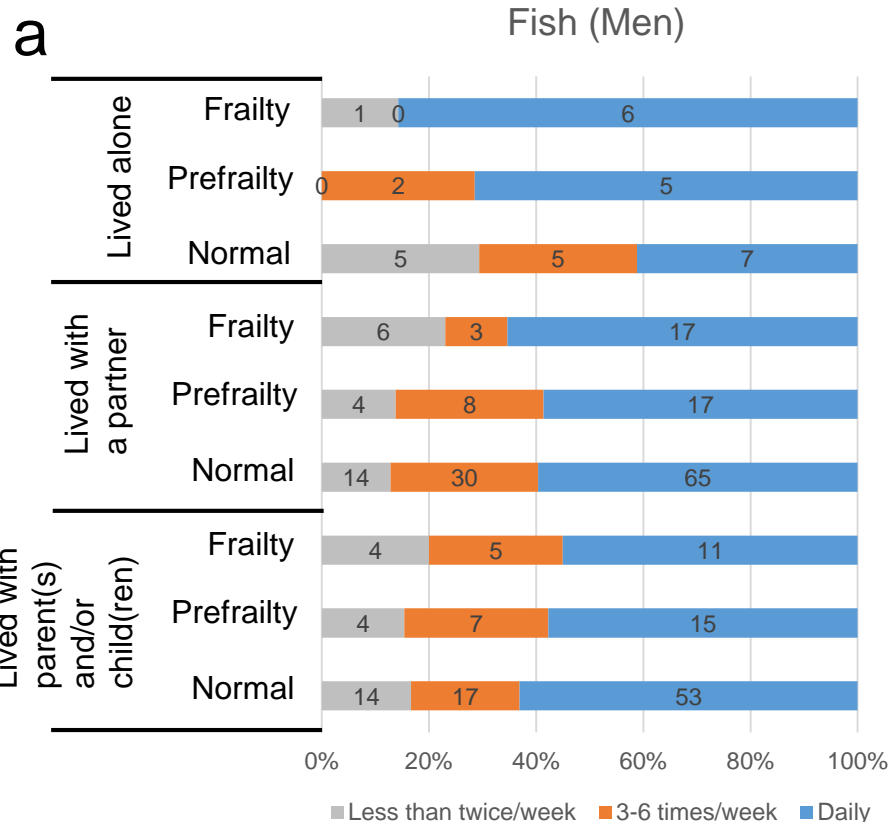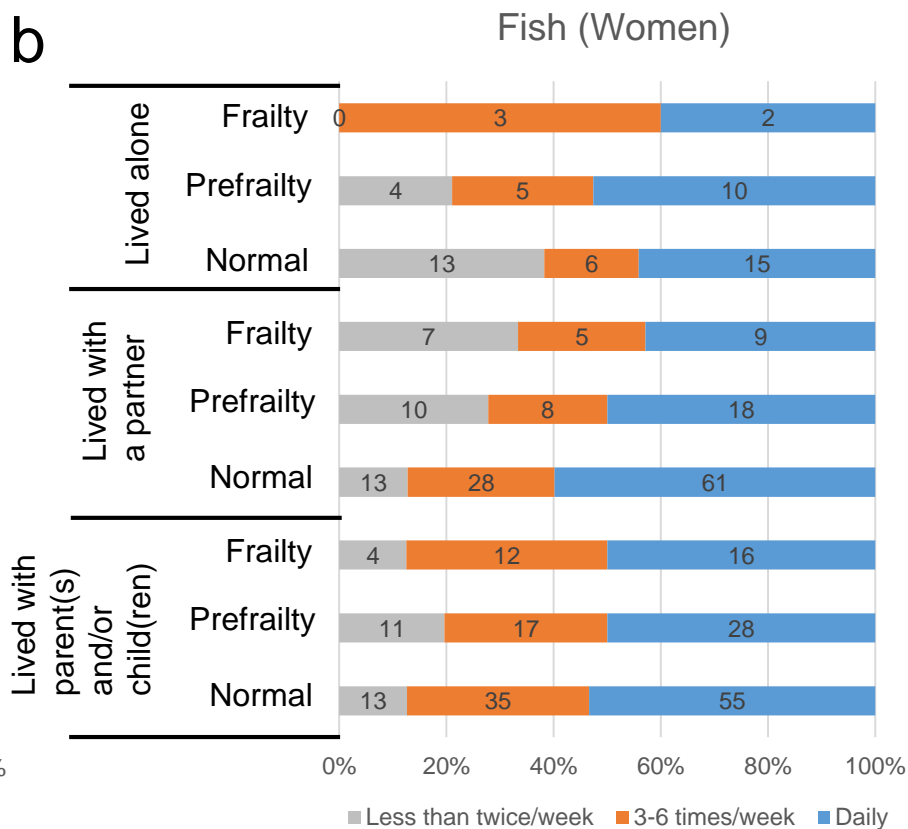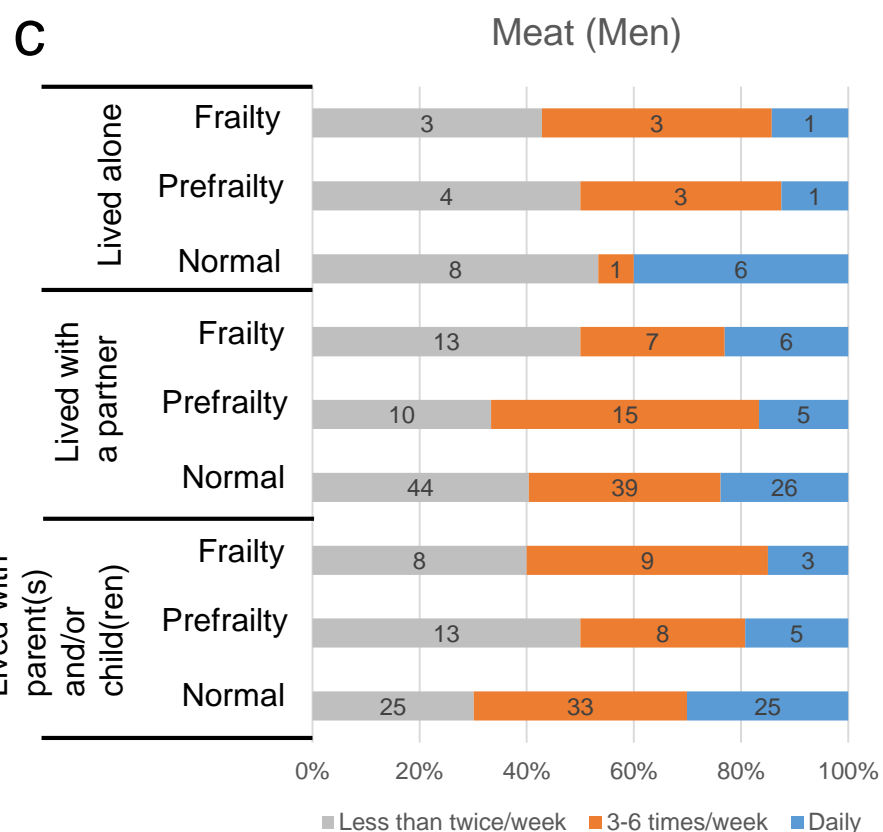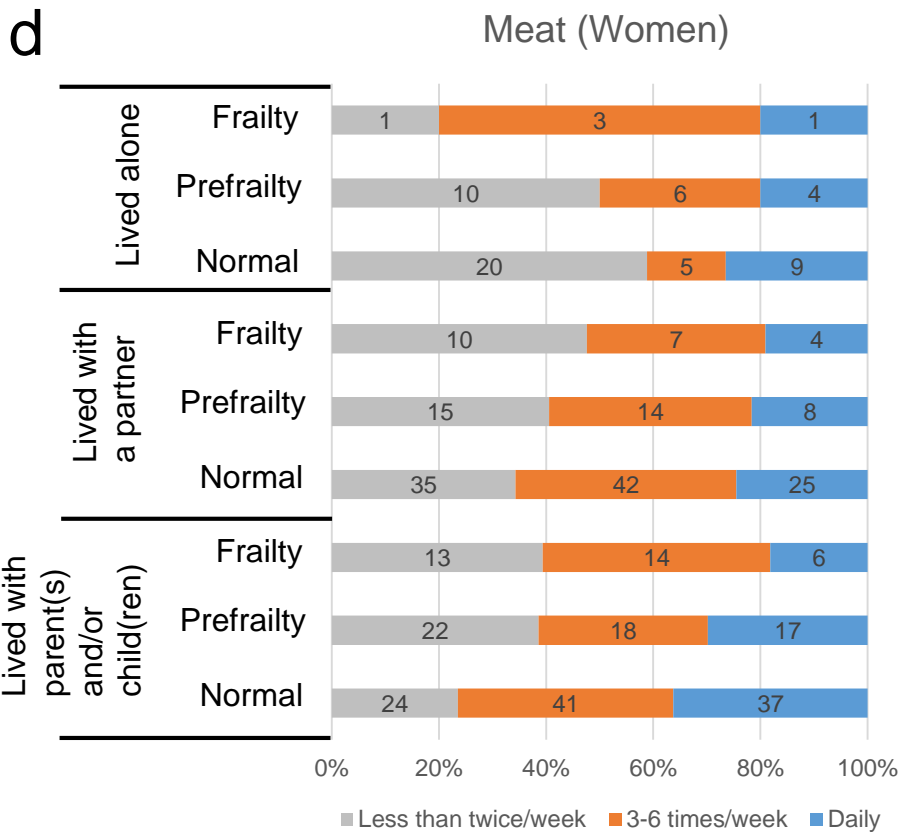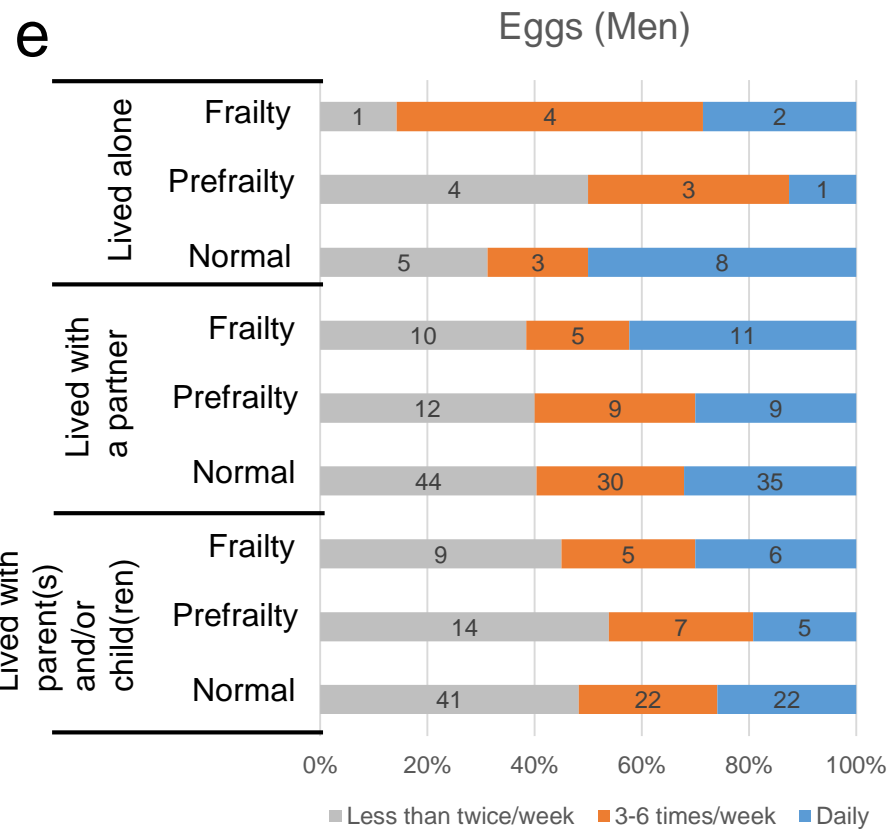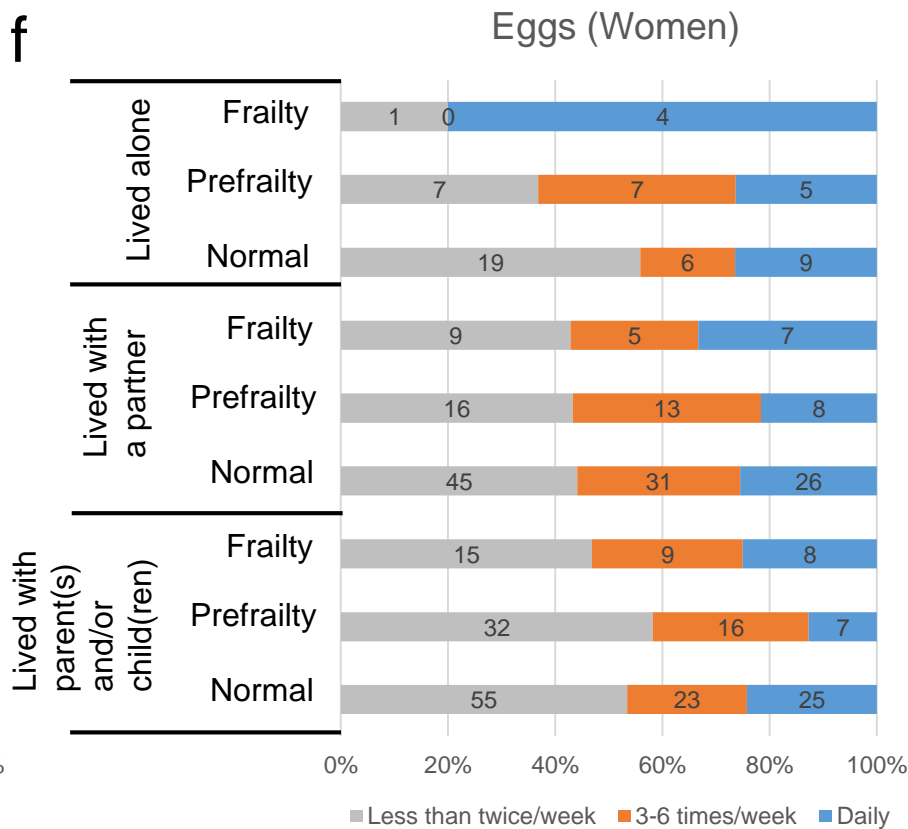

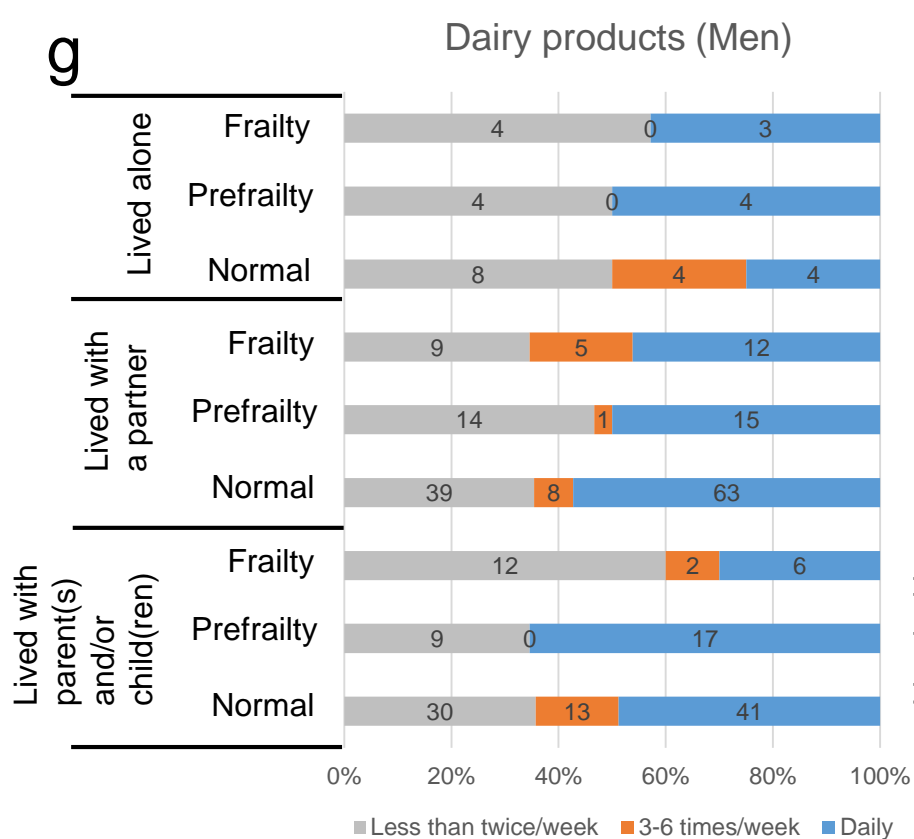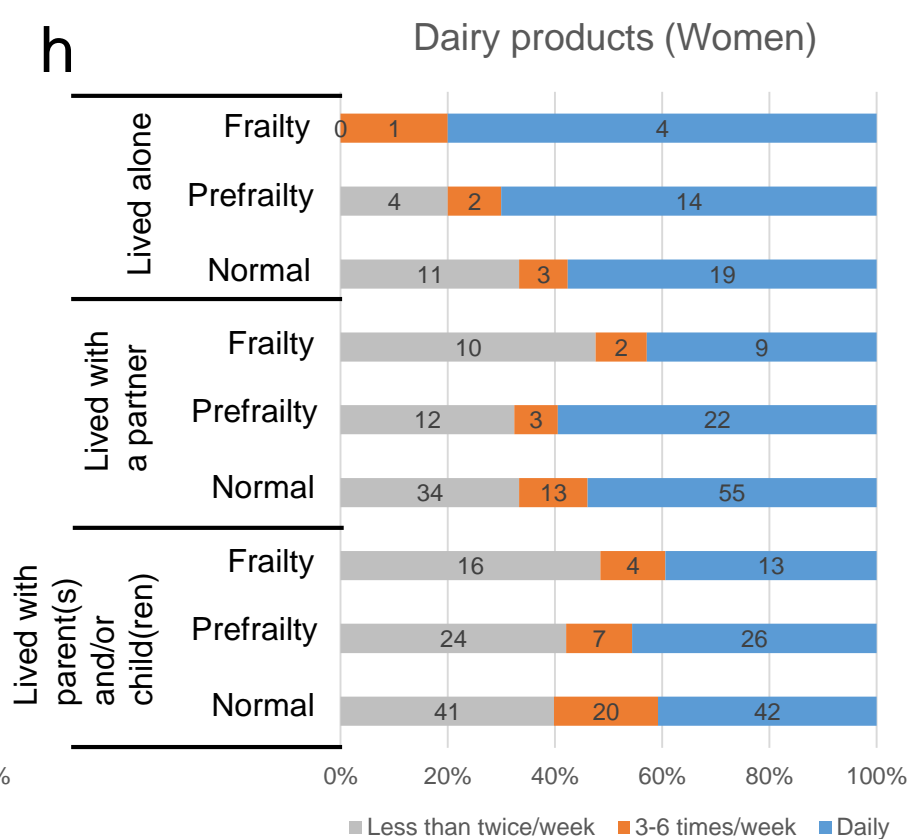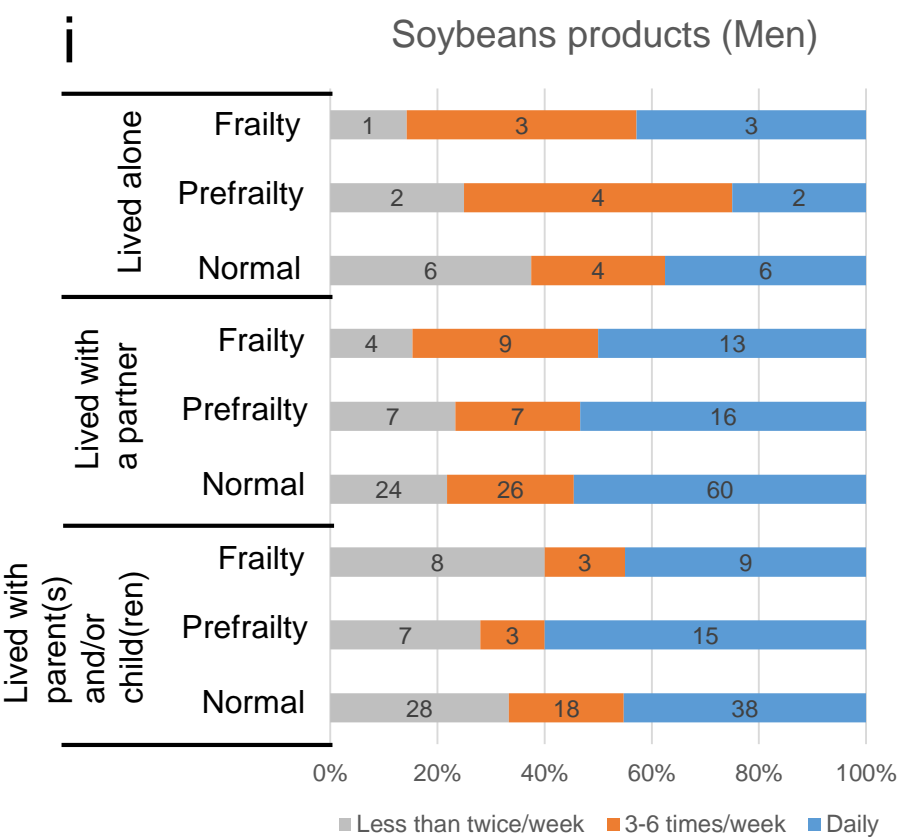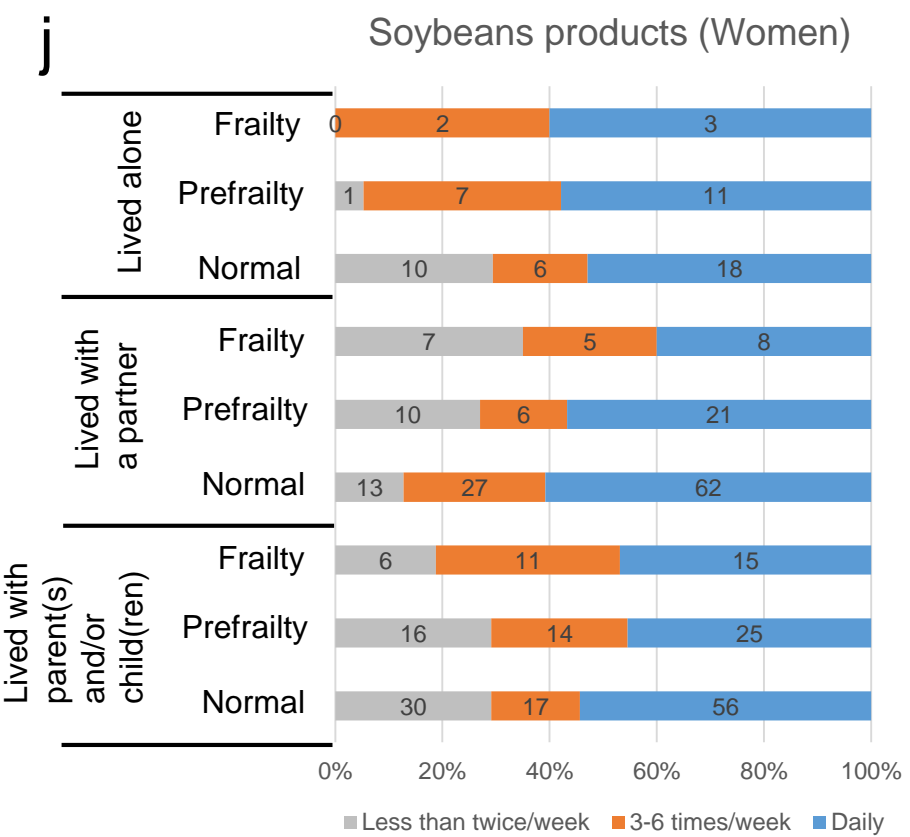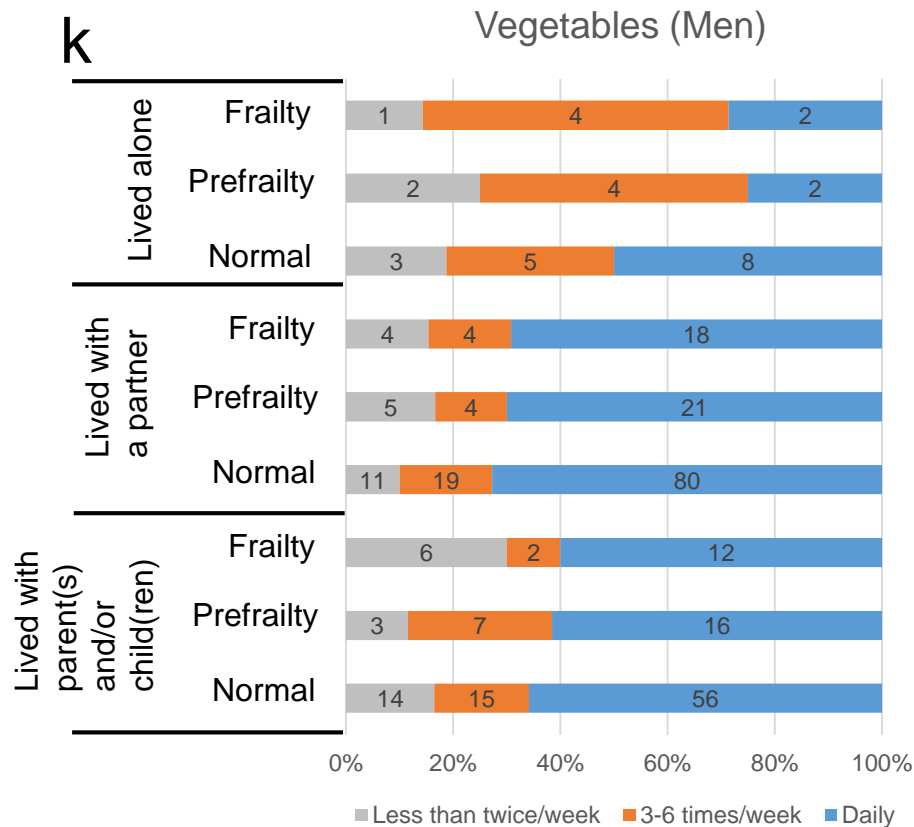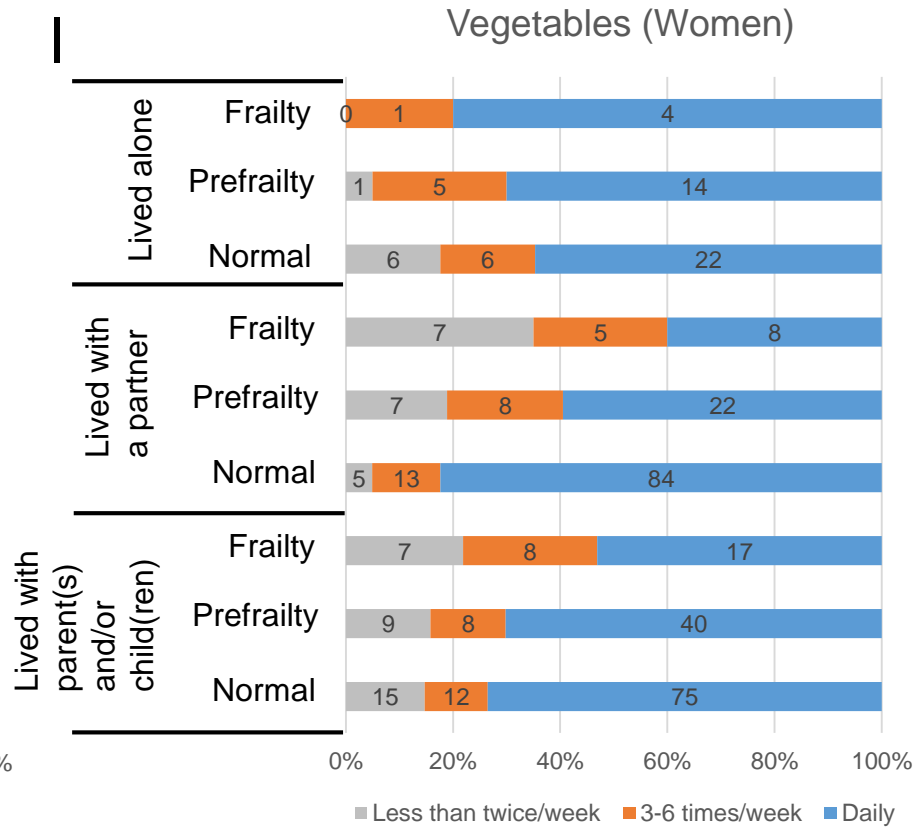

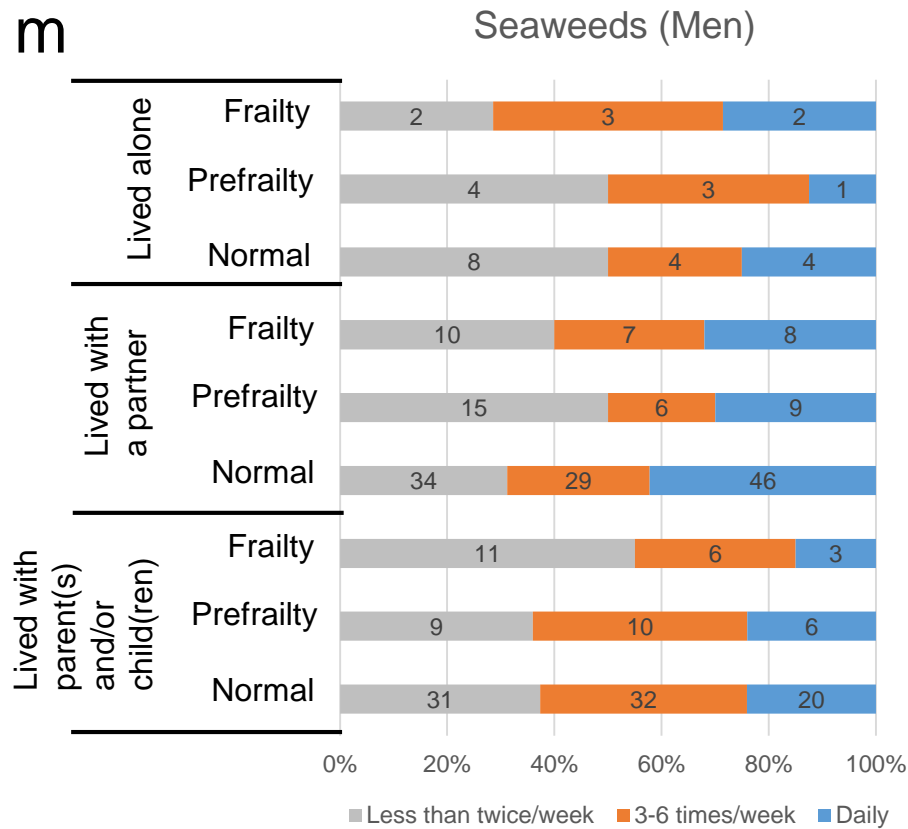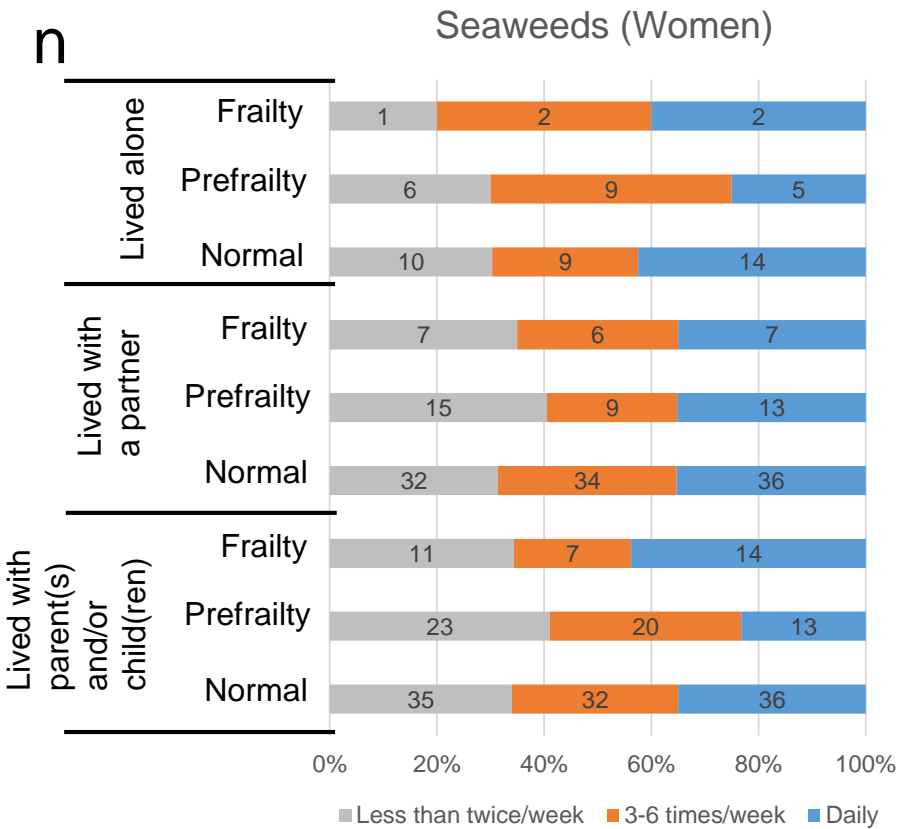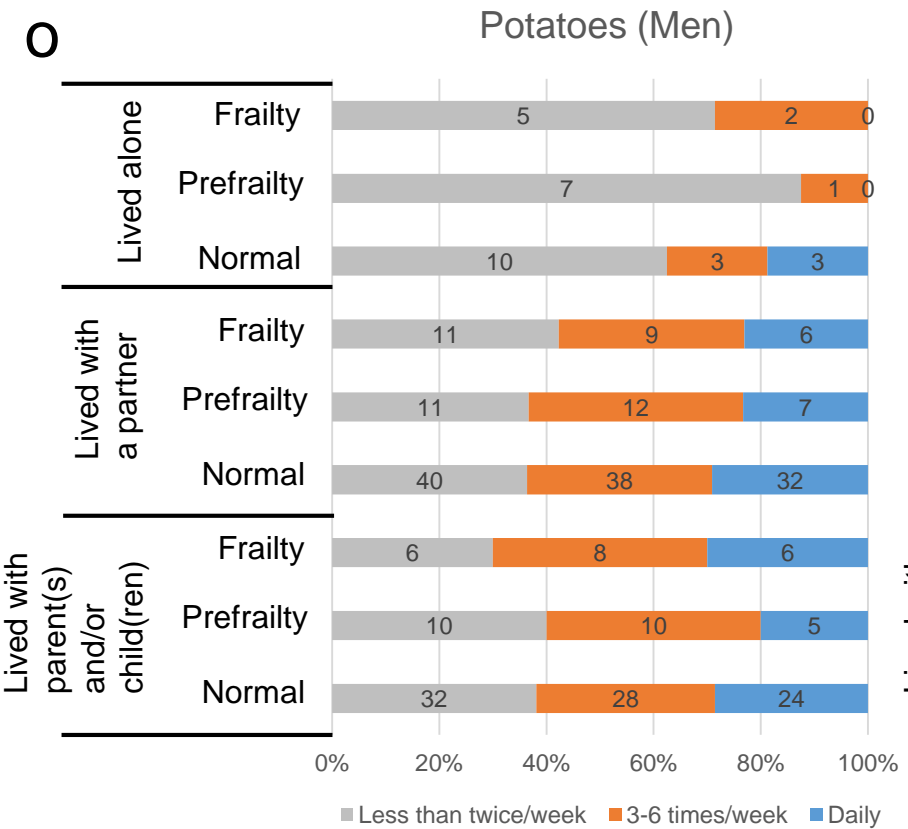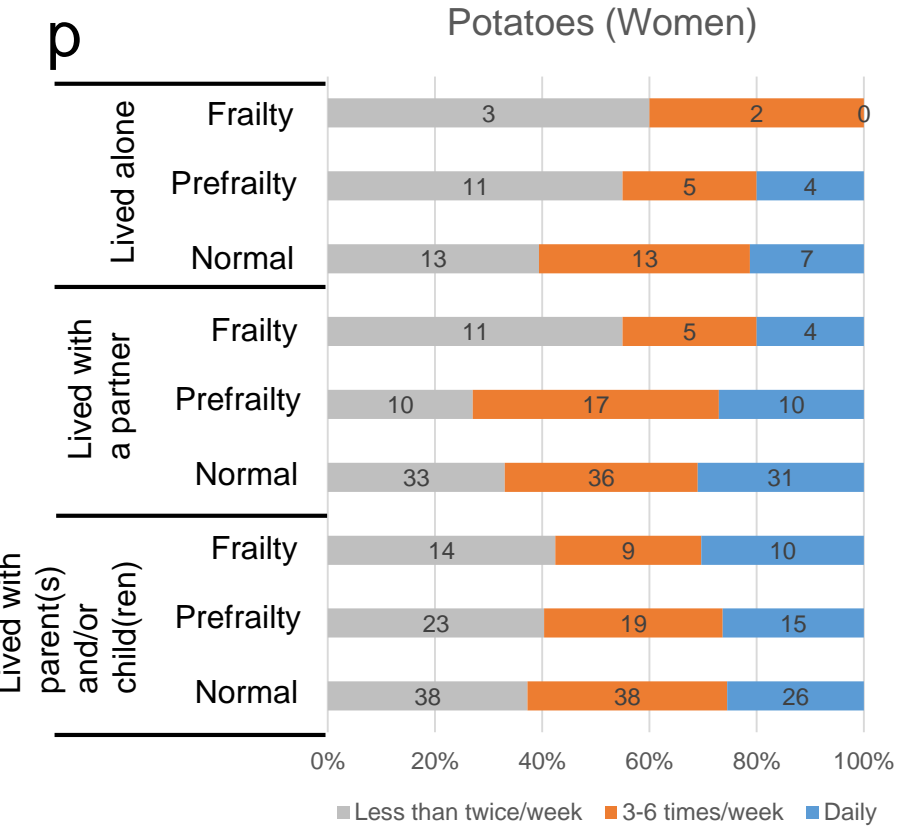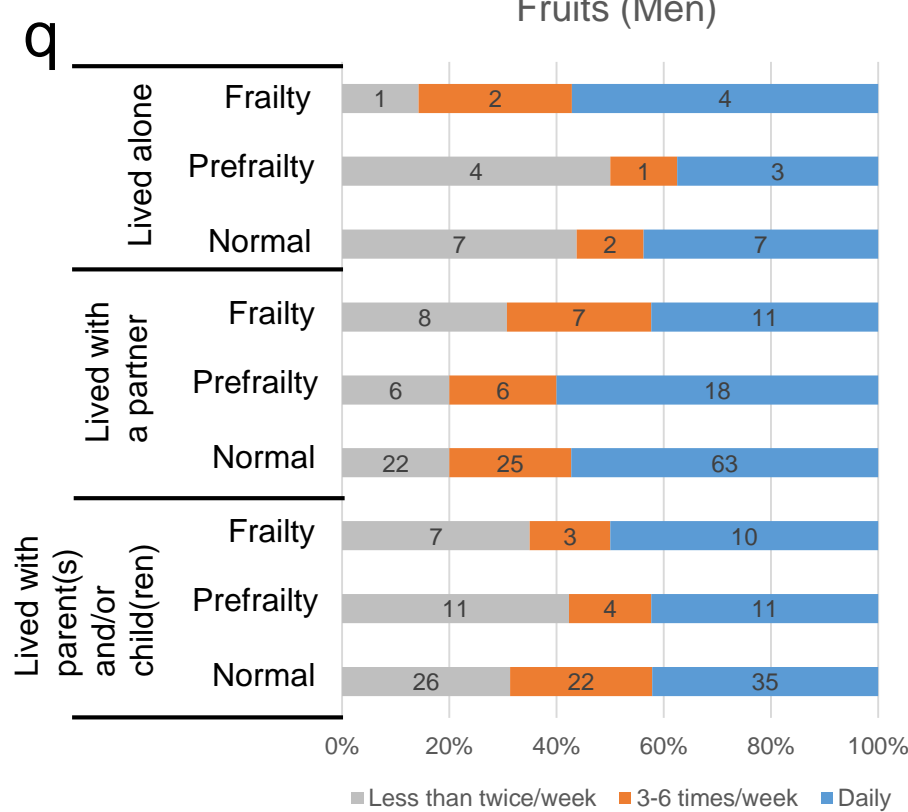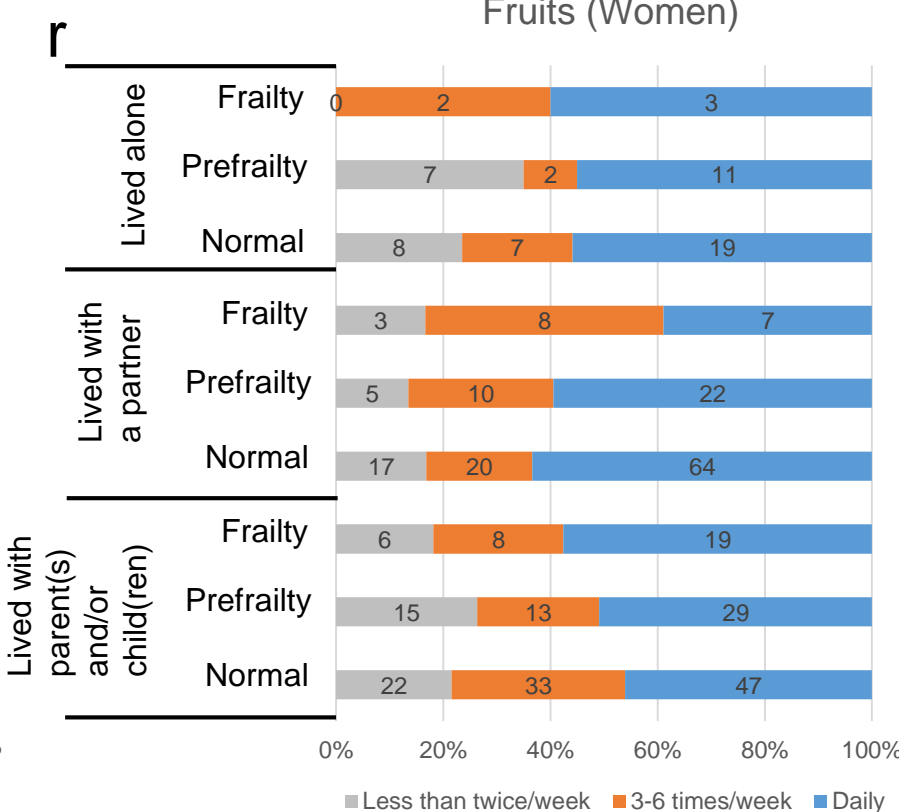

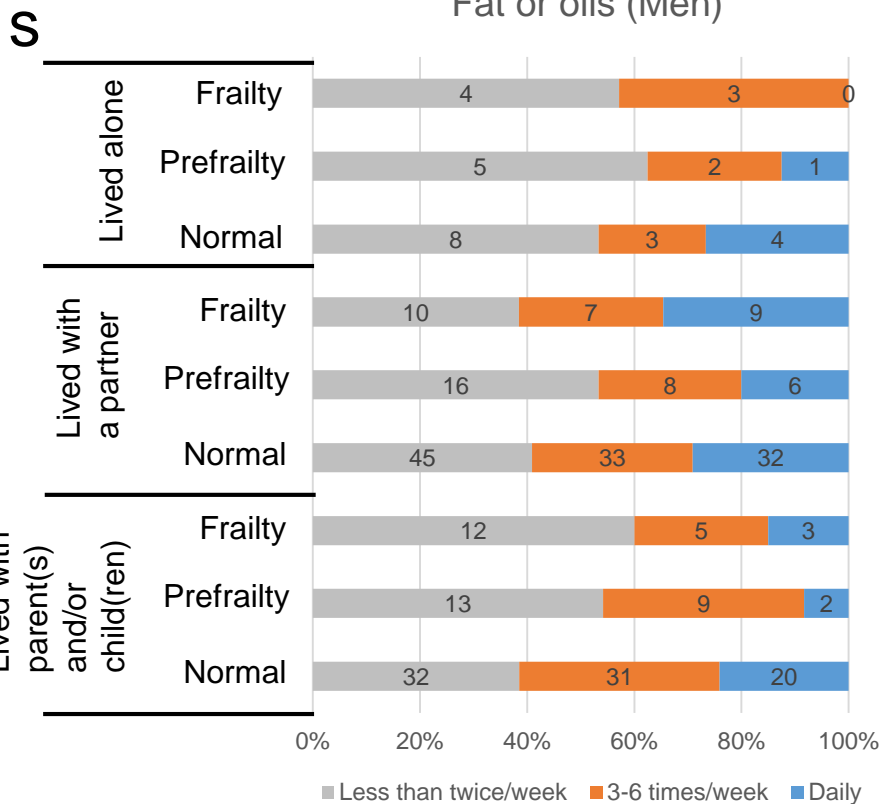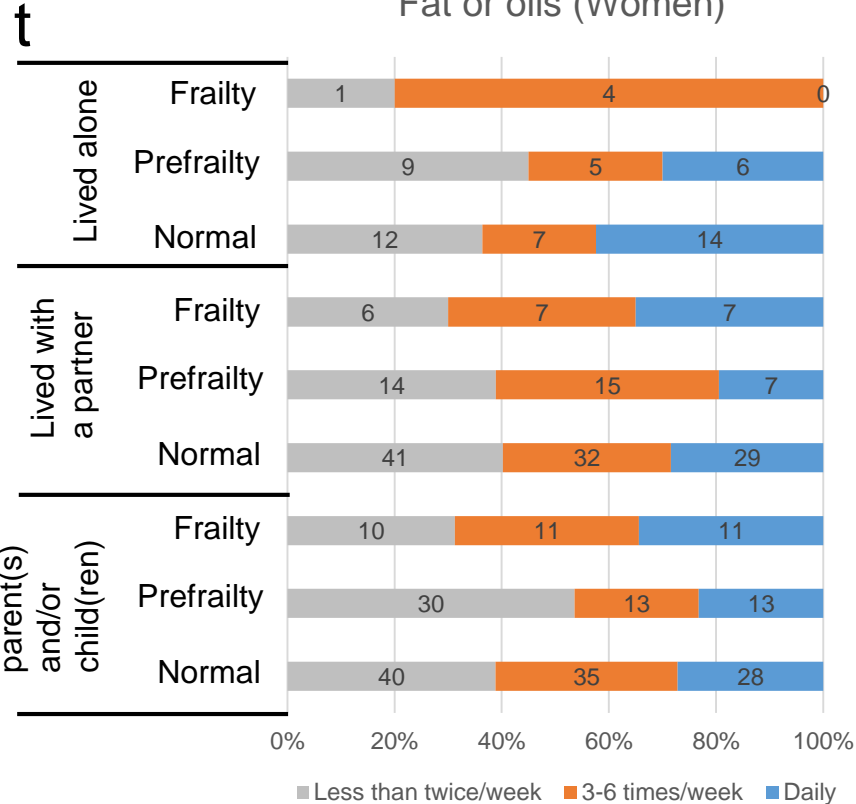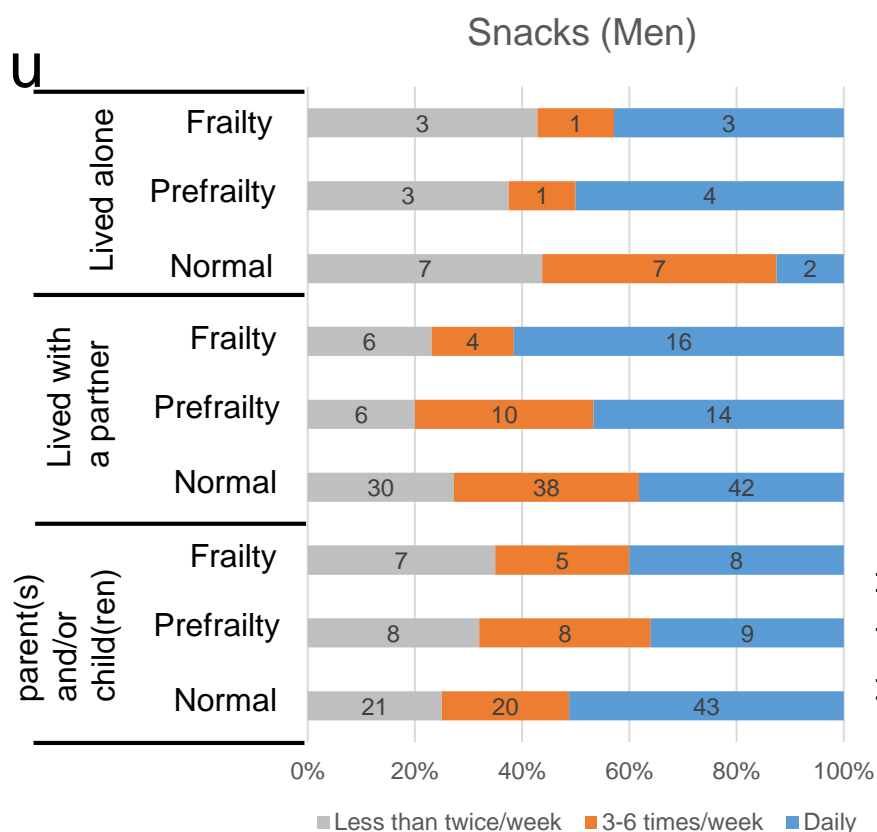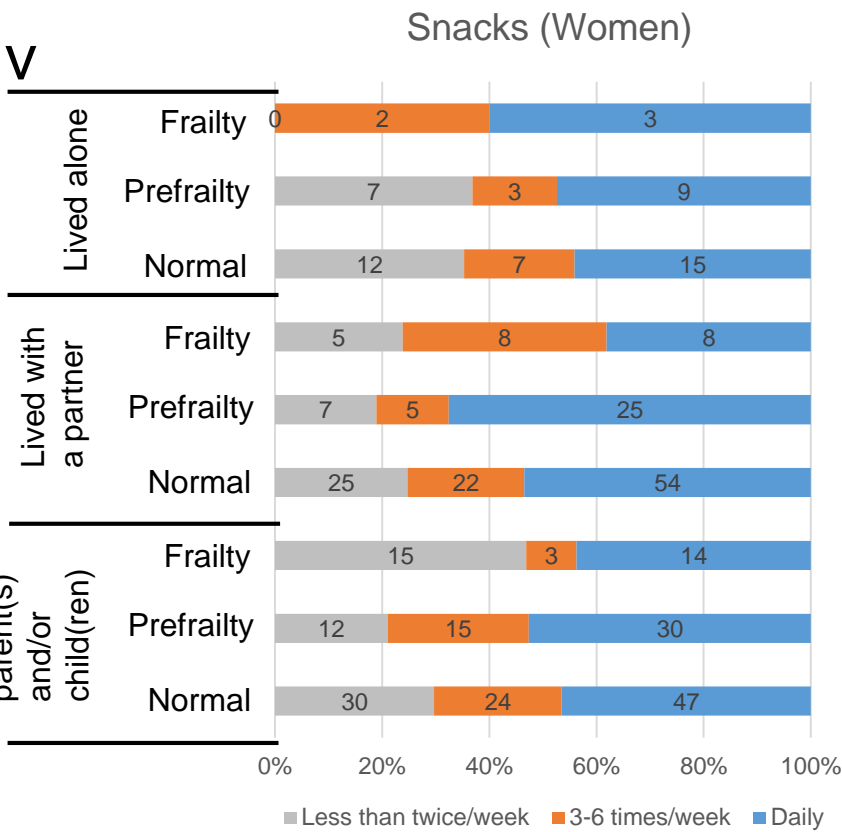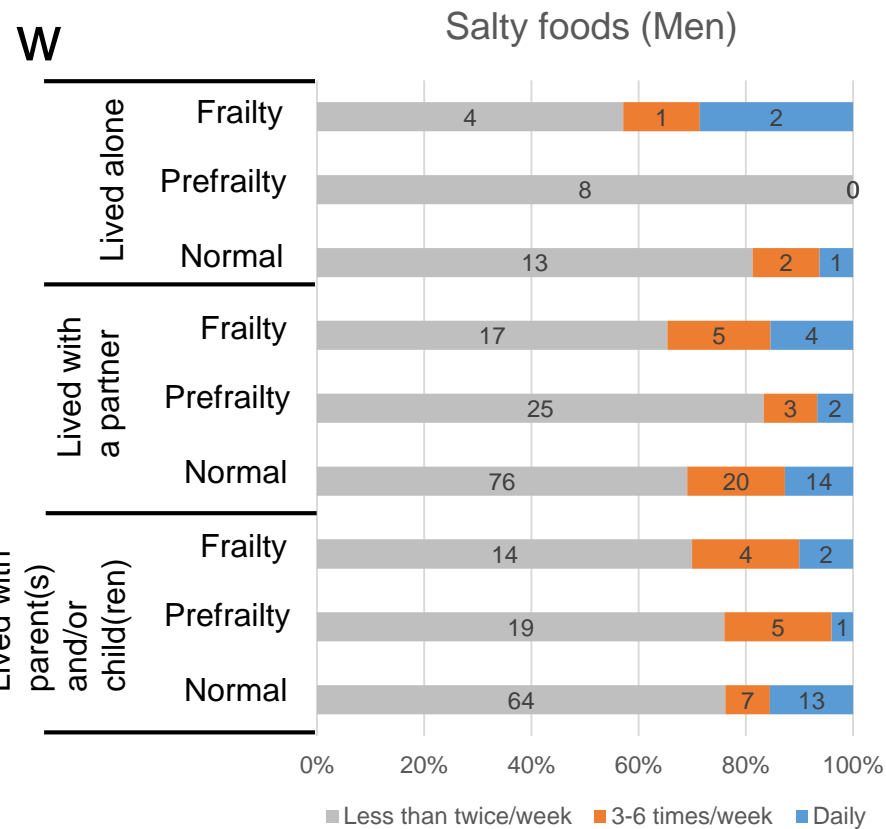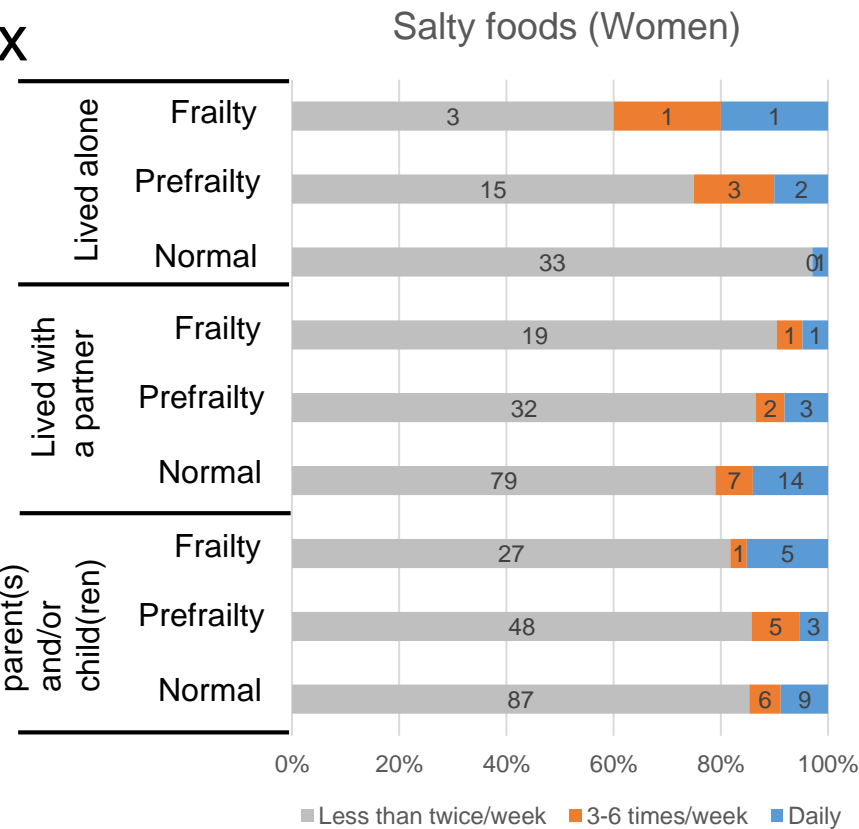

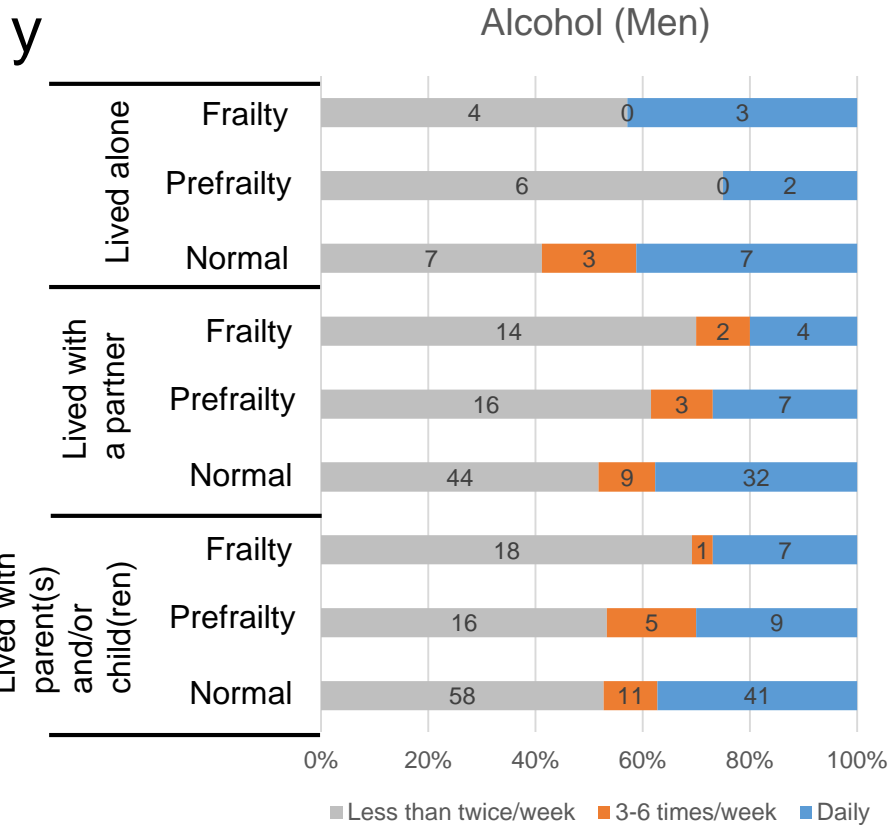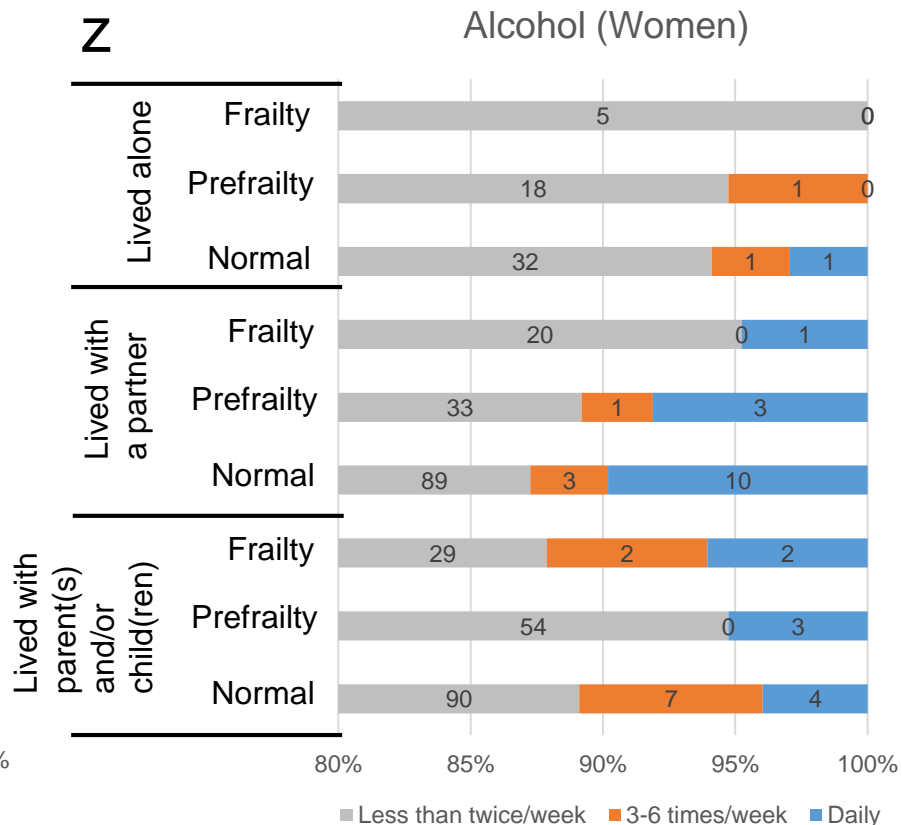

Supplement: Supplementary file 1 — Figure S1. The consumption of each food categories in both men and women. a: Fish (Men), b: Fish (Women), c: Meat (Men), d: Meat (Women), e: Eggs (Men), f: Eggs (Women), g: Dairy products (Men), h: Dairy products (Women), i: Soybeans products (Men), j: Soybeans products (Women), k: Vegetables (Men), l: Vegetables (Women), m: Seaweeds (Men), n: Seaweeds (Women), o: Potatoes (Men), p: Potatoes (Women), q: Fruits (Men), r: Fruits (Women), s: Fat or oils (Men), t: Fat or oils (Women), u: Snacks (Men), v: Snacks (Women), w: Salty foods (Men), x: Salty foods (Women), y: Alcohol (Men), z: Alcohol (Women). The number in each colored bar indicates the number of participants. (PDF 194 kb) [file 12877_2019_1229_MOESM1_ESM.pdf]
