# Supplementary material for: Sex-related differences in the association between frailty and dietary consumption in Japanese older people: a cross-sectional study
Source: BMC Geriatr. 2019 Aug 5;19:211. doi: 10.1186/s12877-019-1229-5 (PMC6683375; doi:10.1186/s12877-019-1229-5)
Supplement: Supplementary file 2 — Table S1. Interaction between sex and dietary consumption according to living arrangement (DOCX 23 kb) [file 12877_2019_1229_MOESM2_ESM.docx]

**Additional file 2: Table S1. Interaction between sex and dietary consumption according to living arrangement**

|  | Lived alone | | Lived with a partner | | Lived with parent (s) and/or children | |
| --- | --- | --- | --- | --- | --- | --- |
|  | F | p-value | F | p-value | F | p-value |
| Fish | 2.917 | 0.060† | 0.879 | 0.416 | 0.087 | 0.917 |
| Meat | 0.656 | 0.522 | 0.101 | 0.904 | 0.066 | 0.936 |
| Eggs | 2.277 | 0.109 | 0.045 | 0.956 | 0.197 | 0.821 |
| Dairy products | 2.497 | 0.089† | 1.269 | 0.283 | 0.378 | 0.686 |
| Soybeans products | 0.105 | 0.901 | 2.856 | 0.059† | 2.258 | 0.106 |
| Vegetables | 1.007 | 0.370 | 4.009 | 0.019* | 0.938 | 0.392 |
| Seaweeds | 0.124 | 0.883 | 0.333 | 0.717 | 0.384 | 0.682 |
| Potatoes | 0.851 | 0.431 | 0.157 | 0.855 | 0.726 | 0.484 |
| Fruits | 0.425 | 0.655 | 0.932 | 0.395 | 0.170 | 0.844 |
| Fat or oil | 0.388 | 0.679 | 0.338 | 0.714 | 1.467 | 0.232 |
| Snacks | 1.950 | 0.149 | 2.289 | 0.103 | 0.762 | 0.467 |
| Salty foods | 1.124 | 0.330 | 0.539 | 0.584 | 1.517 | 0.221 |
| Alcohol | 0.854 | 0.429 | 0.035 | 0.965 | 0.913 | 0.402 |

†p<0.10, *p<0.05, The interactions were calculated by using two-way analysis of variance (ANOVA)
